# Effects of re-challenge with temozolomide in grade 2/3 IDH mutant gliomas at first progression

**DOI:** 10.1007/s11060-025-05087-w

**Published:** 2025-08-20

**Authors:** Lalanthica V. Yogendran, Abhinav Kareddy, Salma O. Abbas, Zachary Scharf, James Patrie, Sohil H. Patel, David Schiff

**Affiliations:** 1 Department of Neurology and Rehabilitation Medicine, University of Cincinnati College of Medicine, 3133 Bellvue Ave, Cincinnati, OH 45219 USA; 2 Department of Neurology, Division of Neuro-Oncology, University of Virginia School of Medicine, 1240 Lee St, 3rd Floor, Charlottesville, VA 22903 USA; 3 Department of Radiology, Division of Neuroradiology, University of Virginia School of Medicine, 1240 Lee St, 3rd Floor, Charlottesville, VA 22903 USA; 4 Department of Public Health Sciences, Division of Biostatistics, University of Virginia School of Medicine, 1240 Lee St, 3rd Floor, Charlottesville, VA 22903 USA; 5 1215 Lee St. Room 6225, Charlottesville, VA 22908-0432 USA

**Keywords:** IDH mutation, Temozolomide, Tumor growth rate, RANO criteria, Glioma

## Abstract

**Background:**

Patients with WHO grade 2 and 3 isocitrate dehydrogenase mutation (IDHmt) gliomas commonly receive temozolomide (TMZ), with or without radiation therapy, as initial therapy. At progression, TMZ is sometimes reinstated despite a paucity of data on effectiveness.

**Methods:**

We reviewed imaging outcomes of patients with WHO 2016 grade II/III IDHmt gliomas re-treated with TMZ at first progression between 2007 and 2019. Tumor growth rates were calculated over the year preceding re-treatment and throughout the re-treatment period, ranging from 3 to 41 months. RANO criteria were utilized to assess TMZ response rate.

**Results:**

15 subjects included six grade II, five grade III oligodendrogliomas, one grade II and three grade III astrocytomas. Median time between completion of the first TMZ course and initiation of re-treatment was 47 months. Median progression-free survival with TMZ re-treatment was 27.4 months and median overall survival was 47.8 months. Mean rate of tumor growth by bidimensional product increased from 0.29 cm^2^ /month, in the year prior to first tumor progression, to 0.47 cm^2^/month during re-treatment, ranging from 3 to 41 months, with monotherapy TMZ. Volumetric mean rate of tumor growth was 1.12 cc/month in the year prior to first tumor progression versus 1.29 cc/month during TMZ re-treatment. Five patients experienced tumor growth rate reduction, of whom 3 patients experienced tumor shrinkage as measured by 2D; 2 of these 3 patients also experienced tumor shrinkage as measured by 3D. There was no radiographic response by RANO criteria.

**Conclusion:**

These findings suggest previously treated, progressive IDHmt gliomas are generally resistant to TMZ, underscoring the need for alternative approaches.

**Supplementary Information:**

The online version contains supplementary material available at 10.1007/s11060-025-05087-w.

## Introduction

Gliomas represent more than half of all malignant primary brain tumor cases and are most common among adults; they include a subgroup with mutations in the isocitrate dehydrogenase (IDH) gene [1–5]. These IDH mutant gliomas, particularly those classified as World Health Organization (WHO) grade 2 and grade 3 contain clinical and molecular features that distinguish them from IDH wild-type gliomas. [2, 3] IDH mutant gliomas generally have a more favorable prognosis and slower progression, but they still present significant therapeutic challenges due to the risk of recurrence [6].

Standard treatment for newly diagnosed IDH mutant grade 2 and grade 3 gliomas involves consideration of surgical resection, radiotherapy (RT), and chemotherapy. Standard of treatment with chemotherapy was established by the results of RTOG 9802 and RTOG 9402 with procarbazine, lomustine and vincristine (PCV). Temozolomide (TMZ), an oral alkylating agent, is a cornerstone of this therapeutic approach introduced for treatment of high grade gliomas and adopted for low grade gliomas in patients who cannot tolerate PCV [7, 8] TMZ has been extensively used in the treatment of various gliomas, due to its ability to cross the blood-brain barrier, oral formulation, efficacy, and relatively favorable side effect profile [9]. TMZ works by methylating DNA, which disrupts the replication of cancer cells, leading to cell death [9]. TMZ has been utilized as an initial strategy to delay RT and in combination with RT as the initial therapy for both grade 2 and grade 3 tumors. The CATNON study unequivocally established the benefits of TMZ in grade III IDH-mutant astrocytomas. ECOG-ACRIN E3F05 also demonstrated the benefit of TMZ in addition to RT in grade II gliomas [10]. TMZ represents a significant advance compared to previous treatment options, although IDH mutation status was not necessarily known or assessed in these earlier studies [11–13].

There is also growing enthusiasm for IDH mutant-targeted treatments, particularly with the recent FDA approval of vorasidenib, although to date the demonstrated benefit has been in patients not previously treated with radiation or cytotoxic chemotherapy. In a recent phase 3 trial, vorasidenib demonstrated significant improvement in median progression-free survival, making it an option for previously untreated patients with IDH mutant low-grade gliomas [14].

Despite TMZ’s initial efficacy, nearly all IDH-mutant gliomas recur, and the clinical benefits of TMZ re-challenge in recurrent IDH-mutant grade 2 and 3 gliomas remain unclear [7, 8, 15–18]. There are limited robust clinical data on TMZ re-challenge, with practices varying widely [7, 18]. Lack of consensus underscores the need for further research and standardized protocols.

Our study aimed to address this gap by evaluating TMZ re-challenge efficacy in recurrent IDH-mutant grade II and III gliomas, assessing radiographic response rates using RANO criteria, and comparing tumor growth rates with 2D and 3D imaging before and during re-treatment. We sought to determine if re-treatment impacts tumor growth dynamics compared to pre-re-treatment rates.

## Methods

### Data collection

This retrospective study was performed at the University of Virginia Medical Center with approval from the institutional review board. Subjects selected for this study were between 18 and 90 years old, had a diagnosis of a grade II/III IDHmt glioma per the 2016 World Health Organization classification, confirmed by tissue and molecular marker pathology. MGMT methylation status was identified for available patients but was not routinely obtained. Subjects included had prior surgery (resection or biopsy), radiation (5 patients), and only received oncologic treatment with TMZ, between 2007 and 2019. Those who received alternative oncologic treatment regimens were excluded. Eligible patients also had radiologic evidence of glioma progression at follow-up, followed by re-treatment with TMZ. Patients without sequential MRI scans available for imaging assessment were excluded. A systematic chart review was conducted to extract data on demographic information, treatment details, imaging outcomes, and follow-up data. Radiologic assessments were based on MRI scans obtained at baseline, during follow-up, and at the time of the next progression. Imaging responses to TMZ were evaluated using the RANO criteria for both 2D and 3D imaging by two board-certified neuroradiologists with 12 and 4 years of experience, respectively. The best response during the initial TMZ treatment was assessed and subsequent responses following re-treatment with TMZ.

### Statistical analyses

Due to the interval censoring aspect of data collection, the cumulative distribution for being “tumor progression free” as a function of time (months) was estimated with the non-parametric interval censored estimator of Wellner and Zahn [19]. Overall survival was estimated with the non-parametric product-limit estimator of Fleming-Harrington [20]. Bidimensional product determined rate of tumor growth prior to first tumor progression and during re-treatment with monotherapy TMZ was estimated with a linear mixed model (LMM) and mean bidimensional product determined tumor growth rate was compared between the two assessment periods by way of a LMM linear contrast of the mean bidimensional product determined tumor growth rate. Volumetric determined rate of tumor growth prior to first tumor progression and during TMZ re-treatment was estimated with a LMM and mean volumetric determined tumor growth rate was compared between the two assessment periods with a LMM linear contrast of the mean volumetric determined tumor growth rate.

## Results

**Fig. 1:**
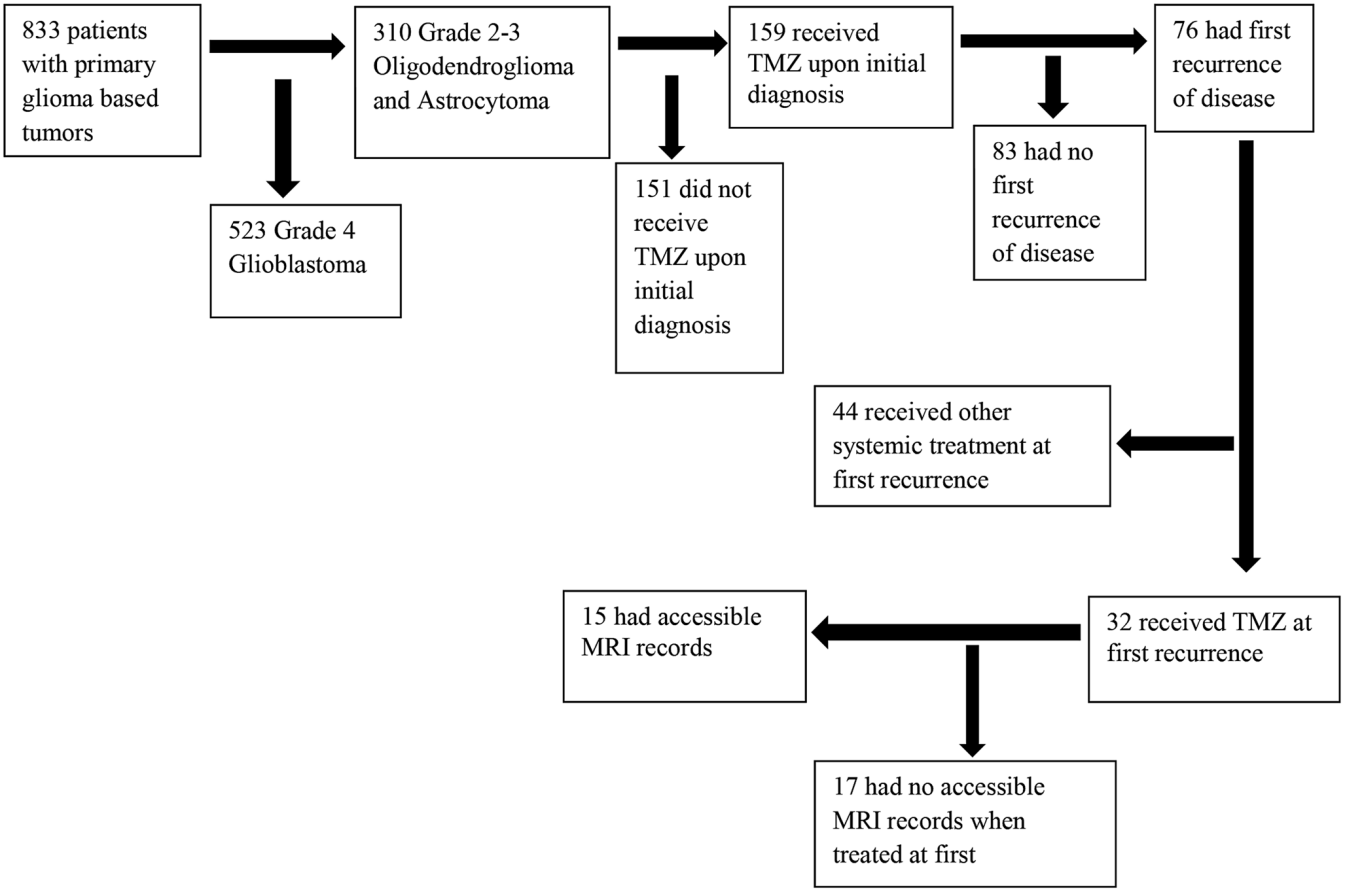
Flowchart of Patient Selection for Final Cohort of Primary Glioma-Based Tumors Treated with Temozolomide

The study cohort consisted of 15 patients (Fig. 1). Eleven patients (73.3%) were male, and all patients were Caucasian and non-Hispanic. The median age was 58.0 years. All 15 patients had grade II or III glioma, astrocytoma or oligodendroglioma according to the 2016 WHO CNS classification. (Table 1). All patients had surgery. 5 patients received initial treatment that included radiation (33.3%), the remaining 10 only received TMZ. The median time between completion of first TMZ course and initiation of re-treatment was 57.4 months. A median number of 11 images were performed at retreatment at frequencies of every 2, 3 and 4 months. At the time of retreatment all patients received monotherapy with TMZ.

**Table 1:** Patient Demographic Data

Demographic Characteristic	
Male gender; n (%)	11 (73.3)
Caucasian Race; n (%)	15 (100%)
Non-Hispanic Ethnicity; n(%)	15 (100%)
Age; median, [IQR], [Range]	58.0 [51.5, 67.0], [31.0, 78.0]
Tumor Type; n (%)	
Grade II Oligodendroglioma Grade III Anaplastic Oligodendroglioma Grade III Anaplastic Astrocytoma Grade II Astrocytoma	6 (40%) 5 (40%) 3 (20%) 1 (6.7%)
Surgery At Diagnosis	
Total Resection Subtotal Resection Biopsy	2 (13.3%) 6 (40%) 7 (46.7%)
Initial treatment at diagnosis:	
TMZ Alone; n (%) Radiotherapy and TMZ	10 (66.7%) 5 (33.3%)
Median time between completion of TMZ course and initiation of re-treatment	47 months (Range: 8-126 months).
Median Karnofsky Performance Status (KPS)	70
O6-Methylguanine-DNA Methyltransferase (MGMT) Methylation Status	Methylated: 2(13.3%) Unmethylated: 2(13.3%) Unknown: 11 (73.3%)
Median Total Number of MRIs done after first reoccurrence	11
Frequency Follow Up with MRIs after first reoccurrence	2 months: 1(6.6%) 3 months: 7 (46.7%) 4 months: 7 (46.7%)

### Tumor progression

**Fig. 2:**
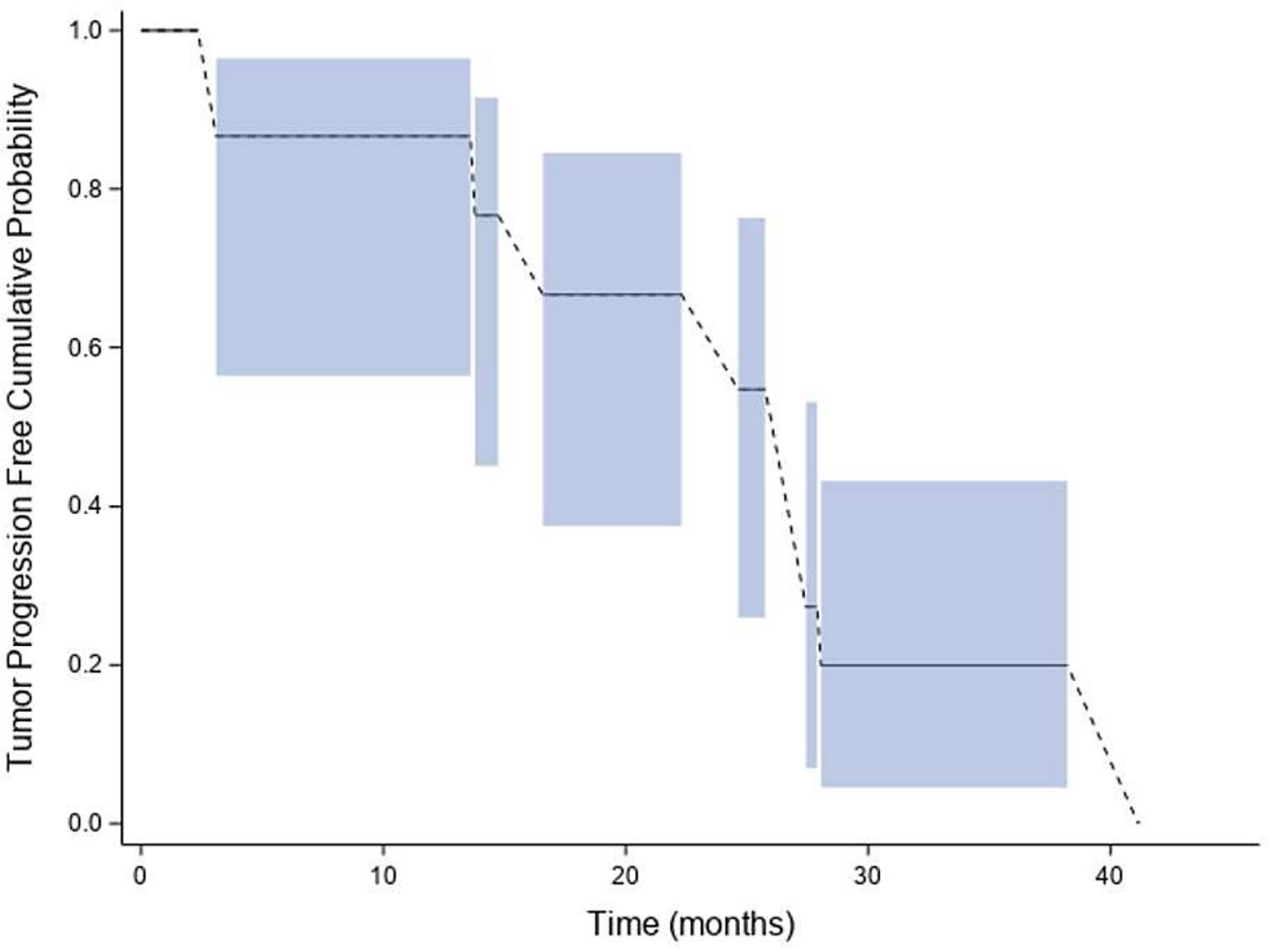
Interval censored “tumor progression free” cumulative distribution function. Shaded areas identify the 95% confidence interval for the cumulative probability for being tumor progression free

**Fig. 3:**
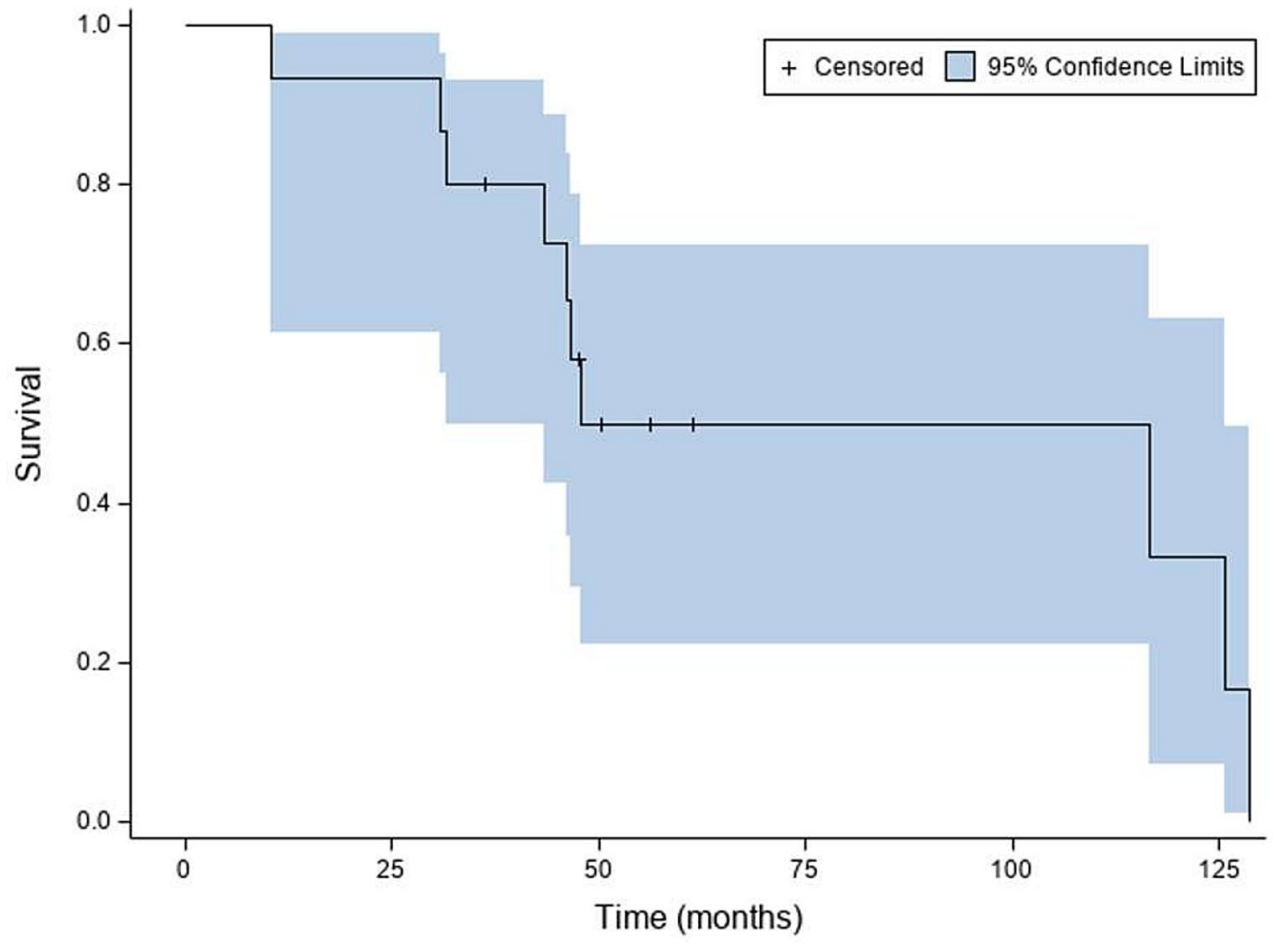
Overall survival cumulative distribution function curve. Shaded areas identify the 95% pointwise confidence interval for the cumulative probability for overall survival

Fig. 2 shows the interval censored “tumor progression free” cumulative probability distribution function as a function of time from the onset of TMZ retreatment as determined by RANO with 2D and 3D measurements. The cumulative probability of being tumor progression free was 0.87 (95% CI: [0.56, 0.97]) from 2.3 months to 3.1 months, and 0.67 (95% CI: [0.38, 0.85]) from 14.7 months to 16.6 months. Median time to tumor progression was 27.4 months (95% CI: [13.8, 28.1] months) (supplemental Table S1). Right censored “overall survival” cumulative probability distribution function is shown in Fig. 3 as a function of time from the onset of TMZ retreatment. At approximately 10 months from the onset of TMZ re-treatment, the cumulative probability for survival was 0.93 (95% CI: [0.61, 0.99]), while at approximately 30 months from the onset of TMZ retreatment, the cumulative probability for survival was 0.87 (95% CI: [0.56, 0.97]. Median survival from the onset of TMZ re-treatment was 47.8 months (95% CI: [31.5, 125.6] months) (supplemental Table S2).

**Table 2:** Astrocytoma vs. Oligodendroglioma comparison of mean change Δ _(After– Prior)_ in 2D and 3D tumor progression rate

Comparison (Astrocytoma– Oligodendroglioma)	Difference in Δ in Tumor Progression Rate (After– Prior)	Lower 95% Confidence Limit	Upper 95% Confidence Limit	*P*-value
2D	0.329 cm^2^/month	-1.553	2.211	0.636
3D	-0.289 cc/month	-5.830	5.251	0.900

### 2D tumor progression rate

**Fig. 4:**
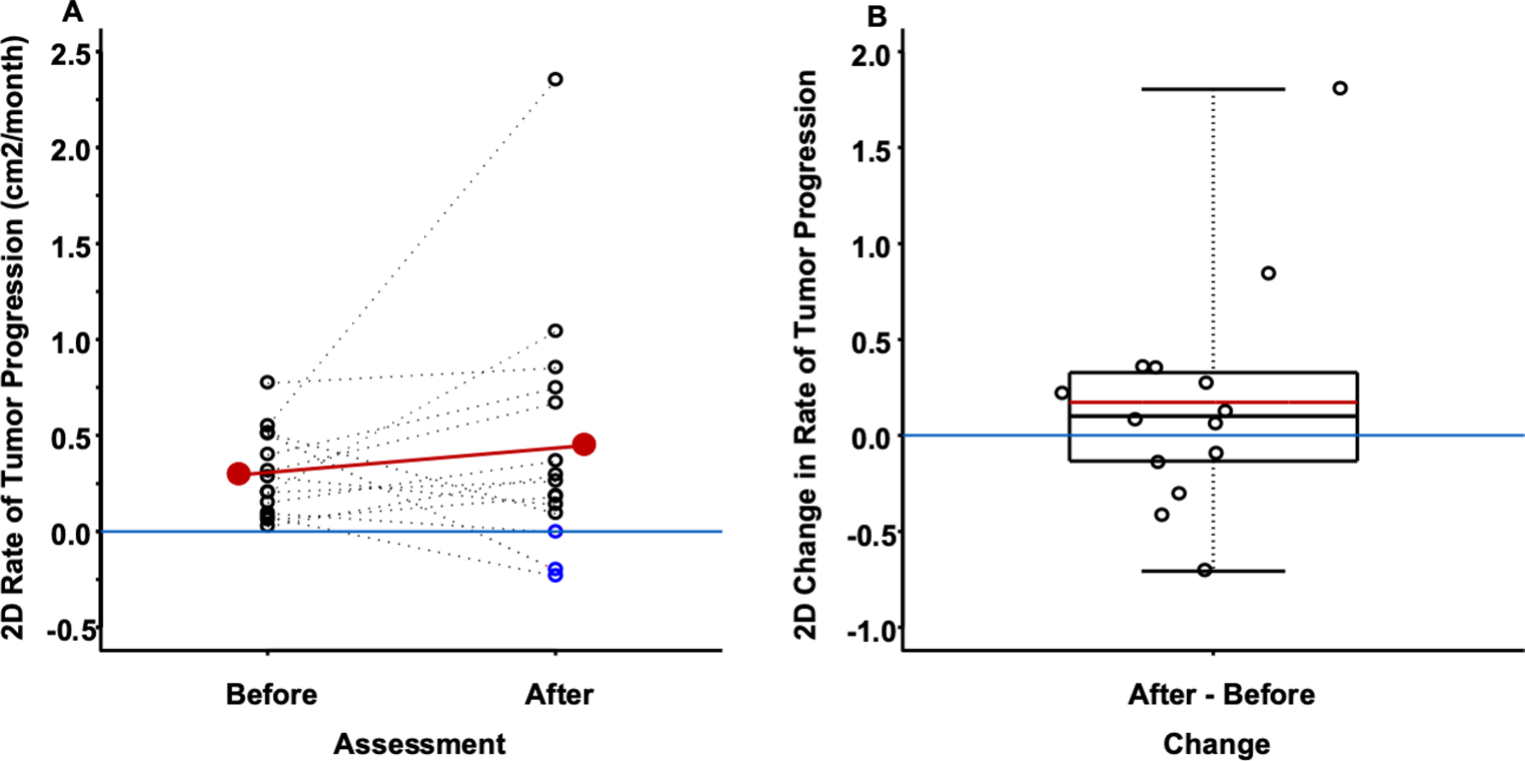
Before and after 1^st^ tumor progression 2D rates of change in tumor progression (cm^2^/month) (**A**), and the 2D changes in tumor progression rate from before 1^st^ tumor progression to after 1^st^ tumor progression (**B**). The red line in A connects the before and after 1^st^ tumor progression 2D mean tumor progression rates. The blue horizontal line in A differentiates positive versus negative rates of 2D tumor progression. Blue circles in A identify tumor shrinkage. The red horizontal line in B is the mean of the distribution of 2D tumor progression rate changes from before 1^st^ tumor progression to after 1^st^ tumor progression, and the black horizontal line in B is the median of the distribution of 2D tumor progression rates changes from before 1^st^ tumor progression to after 1^st^ tumor progression, and the blue horizontal line in B differentiates positive versus negative tumor progression changes

15 patients had pre- and post- first tumor progression rate measurements. Pre- and post-first tumor 2D progression rates are summarized in Fig. 4a-b. Mean 2D tumor progression growth rate was 0.294 cm²/month (95% CI: [0.165, 0.424 cm²/month], *p* < 0.001) before the first progression and increased to 0.466 cm²/month (95% CI: [0.084, 0.849 cm²/month], *p* = 0.021) during TMZ re-treatment (supplemental Table S3). The mean change in 2D tumor progression growth rate was 0.172 cm²/month (95% CI: [-0.225, 0.569], *p* = 0.371). 10 out of 15 patients experienced a tumor growth rate increase during TMZ retreatment. The remaining five patients experienced tumor growth rate reduction of whom three patients experienced tumor shrinkage as measured by 2D (2.6–32.6%). Utilizing RANO criteria, no radiographic partial or complete responses to TMZ re-treatment were detected. There was no difference in the rate of tumor progression for all oligodendrogliomas combined (*n* = 11), and all astrocytomas combined (*n* = 4) by 2D tumor measurements (Table 2).

### 3D tumor progression rate

**Fig. 5:**
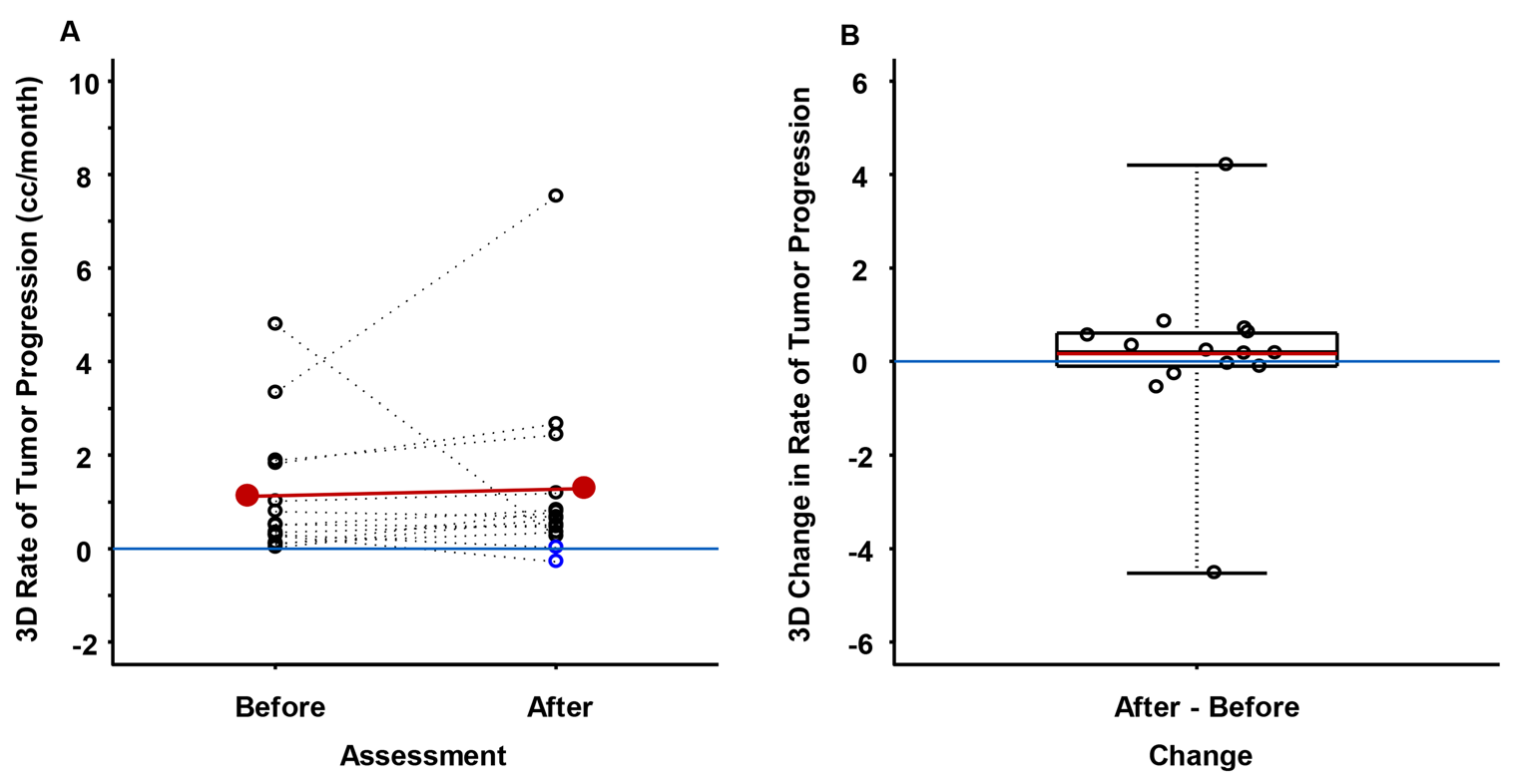
Before and after 1^st^ tumor progression 3D rates of change in tumor progression (cc/month) (**A**), and the 3D changes in tumor progression rate from before 1^st^ tumor progression to after 1^st^ tumor progression. The red line in A connects the before and after 1^st^ tumor progression 3D mean tumor progression rates. The blue horizontal line in A differentiates positive versus negative rates of tumor progression. Blue circles in A identify tumor shrinkage. The red horizontal line in B is the mean of the distribution of 3D tumor progression rate changes from before 1^st^ tumor progression to after 1^st^ tumor progression, and the black horizontal line in B is the median of the distribution of 3D tumor progression rates changes from before 1^st^ tumor progression to after 1^st^ tumor progression, and the blue horizontal line in B differentiates positive versus negative tumor progression changes

Pre- and post-first tumor 3D progression rates are summarized in Fig. 5a-b. Mean 3D tumor growth rate was 1.124 cc/month (95% CI: [0.314, 1.933 cc/month], *p* < 0.001) prior to first progression and increased to 1.287 cc/month (95% CI: [0.147, 2.428 cc/month], *p* = 0.030) during TMZ re-treatment (supplemental Table S4). Difference in mean 3D tumor growth rate was 0.164 cc/month and was not statistically significant (*p* = 0.802). The same five patients from the 2D measurements also experienced 3D tumor growth rate reduction, of whom two patient experienced tumor shrinkage as measured by 3D (8.2–26.8%). Utilizing RANO criteria, no radiographic partial or complete responses to TMZ re-treatment were detected. As with 2D measurements, by 3D measurements there was no difference in the rate of tumor progression for all oligodendrogliomas combined (*n* = 11), and all astrocytomas combined (*n* = 4) (Table 2).

## Discussion

Our study provides insights into the efficacy of TMZ re-treatment for recurrent IDH mutant grade 2 and grade 3 gliomas. The majority of patients (10 out of 15) had tumors with increasing growth rates after TMZ retreatment. Three patients had minor degrees of tumor shrinkage by 2-dimensional and 1 patient had tumor shrinkage by volumetric measurements, and none of these changes qualified as a partial response meeting RANO criteria. Analysis of 2D and 3D tumor growth rates by type of glioma did not differ between the oligodendroglioma group and the astrocytoma group. Overall, our findings indicate that TMZ re-treatment in our patients did not significantly alter tumor growth rates or lead to meaningful radiographic responses compared to the time leading to first progression. These highlight that recurrent IDH mutant gliomas exhibit resistance to TMZ re-treatment as assessed using RANO criteria.

Although only 4 patients had identified MGMT status, the significance of MGMT status in this population is unproven, as the CATNON study failed to show independent prognostic significance of MGMT in IDH mutant grade III astrocytomas [13]. A key factor contributing to TMZ resistance is the development of a hypermutator phenotype, which occurs in a significant fraction of patients treated with TMZ. For TMZ to be effective, intact mismatch repair is essential, and mutations in this pathway can render the treatment ineffective. The hypermutator phenotype has been observed in as many as 57% of TMZ-treated IDH mutant lower-grade gliomas at recurrence [21]. Although our study did not include biopsies at recurrence, it is likely that some of our patients had developed this hypermutator state, contributing to the lack of treatment efficacy. The prevalence of TMZ-induced hypermutation, particularly in recurrent low-grade gliomas, is associated with high-grade transformation, worse prognosis, and increased risk of multifocal disease [22].

This study fills a literature gap regarding the effectiveness of TMZ upon recurrence. While TMZ has shown efficacy as an initial treatment for IDH mutant gliomas, its utility in the context of recurrence has been less clearly defined [4, 7, 8, 15–17, 23, 24]. Our detailed analysis suggests potential drawbacks to re-administering TMZ, which may help inform clinical decisions regarding treatment options for recurrent IDH mutant gliomas.

These findings underscore the need for alternative therapeutic approaches. Data on second-line treatments for tumors progressing despite initial radiotherapy and TMZ is sparse. Studies on nitrosoureas predate molecular IDH testing: Triebels et al. 2004 reported a 17% radiographic response rate to PCV in patients with oligodendrogliomas, though responses were not durable. Similarly, Chamberlain et al. 2015 found a 5.7% response rate and a median PFS of 4.5 months with CCNU in anaplastic astrocytoma patients after TMZ failure. Approximately two-thirds of these patients had IDH mutations, but specific outcomes for IDH-mutant subgroups were not detailed.

The use of IDH mutant-targeted treatments including vorasidenib provides an alternative treatment option. However, for enhancing tumors and patients who are not treatment-naive, the evidence remains limited. Data on their activity in previously treated or enhancing tumors are still sparse, and further research is needed to better understand their role in these subgroups.

This study has several limitations. The small sample size of 15 patients limits the statistical power, potentially obscuring subtle effects of TMZ re-treatment. As noted above, it is likely that some of our patients had acquired mismatch repair mutations rendering TMZ ineffective; a larger study of patients with intact mismatch repair might have identified responders. Additionally, the retrospective design introduces inherent biases and limits control over confounding variables. Data from a single institution may affect the external validity of the results. Larger, prospective studies are needed to validate our findings and further explore novel therapeutic strategies. Such research is essential for advancing the treatment of recurrent IDH mutant gliomas and improving patient outcomes.

In summary, this study underscores the limitations of TMZ re-treatment and highlights the need for developing and evaluating new therapeutic options. These findings will inform clinical practice and guide future research, aiming to enhance the management and outcomes for patients with recurrent IDH mutant gliomas.

## Electronic supplementary material

Below is the link to the electronic supplementary material.

Supplementary Material 1

## Data Availability

No datasets were generated or analysed during the current study.
