# Supplementary material for: Effects of re-challenge with temozolomide in grade 2/3 IDH mutant gliomas at first progression
Source: J Neurooncol. 2025 Aug 20;175(3):1147–54. doi: 10.1007/s11060-025-05087-w (PMC12511160; doi:10.1007/s11060-025-05087-w)
Supplement: Supplementary file 1 — Supplementary Material 1 [file 11060_2025_5087_MOESM1_ESM.docx]

**Supplemental Table S1.** Interval censored tumor progression free cumulative distribution function (CDF) estimated by the non-parametric cumulative distribution function estimator of Wellner and Zahn (1997).

| Time Interval | | CDF Estimation | | 95% Confidence Interval | |
| --- | --- | --- | --- | --- | --- |
| Left  Boundary  (months) | Right  Boundary  (months) | Tumor Progression Free  Cumulative Probability | Standard  Error | Tumor Progression Free  Cumulative Probability  Lower CL | Tumor Progression Free  Cumulative Probability  Upper CL |
| 2.3 | 3.1 | 0.87 | 0.09 | 0.56 | 0.97 |
| 13.6 | 13.8 | 0.77 | 0.11 | 0.45 | 0.92 |
| 14.7 | 16.6 | 0.67 | 0.12 | 0.38 | 0.85 |
| 17.9 | 18.0 | 0.67 | 0.12 | 0.38 | 0.85 |
| 22.3 | 24.6 | 0.55 | 0.14 | 0.26 | 0.76 |
| 25.8 | 27.4 | 0.27 | 0.13 | 0.07 | 0.53 |
| 27.9 | 28.1 | 0.20 | 0.11 | 0.05 | 0.43 |
| 38.2 | 41.2 | 0.00 | 0.00 |  |  |
|  |  | Median Time to Tumor  Progression (months) | 95% CI |  |  |
|  |  | 27.4 | [13.8, 28.1] |  |  |

CL = confidence limit.

CI = confidence interval.

**Supplemental Table S2.** Overall survival cumulative distribution function (CDF) estimated by the product-limit estimator of Fleming-Harrington (1991) .

| Time (months) | Overall Survival Cumulative  Distribution Function  CDF Estimate | Lower 95% CI | Upper 95% CL |
| --- | --- | --- | --- |
| 0 | 1.00 | 1.00 | 1.00 |
| 10.5 | 0.93 | 0.61 | 0.99 |
| 30.8 | 0.87 | 0.56 | 0.97 |
| 31.4 | 0.80 | 0.50 | 0.93 |
| 43.3 | 0.73 | 0.43 | 0.89 |
| 46.2 | 0.66 | 0.36 | 0.84 |
| 46.5 | 0.58 | 0.29 | 0.79 |
| 47.8 | 0.50 | 0.22 | 0.72 |
| 116.5 | 0.33 | 0.07 | 0.63 |
| 125.6 | 0.17 | 0.01 | 0.50 |
| 128.6 | 0 |  |  |
|  | Median Survival Time (months) | 95% CI |  |
|  | 47.8 | [31.5, 125.6] |  |

**Supplemental Table S3**. Linear mixed model summary for comparing the mean change in 2D tumor progression growth rate (cm^2^/month) from prior to 1^st^ tumor progression growth rate to after 1^st^ tumor progression.

| Tumor Progression Rate  Assessment | Mean Tumor Progression Rate (cm^2^/month) | Lower 95% CL | Lower 95% CL | P-value† |
| --- | --- | --- | --- | --- |
| Prior to 1^st^ Progression | 0.294 | 0.165 | 0.424 | <0.001 |
| After 1^st^ Progression | 0.466 | 0.084 | 0.849 | 0.021 |
| Change Δ | Mean Change in Tumor Progression Rate (cm^2^/month) | Lower 95% CL | Lower 95% CL | P-value |
| Δ_(After – Prior)_ | 0.172 | -0.225 | 0.569 | 0.371 |

CL = confidence limit.

† P-value is for the testing of the null hypothesis that the mean tumor progression rate is equal to 0.

**Supplemental Table S4**. Linear mixed model summary for comparing the mean change in 3D tumor progression growth rate (cc/month) from prior to 1^st^ tumor progression growth rate to after 1^st^ tumor progression.

| Tumor Progression Rate  Assessment | Mean Tumor Progression Rate (cm^2^/month) | Lower 95% CL | Lower 95% CL | P-value† |
| --- | --- | --- | --- | --- |
| Prior to 1^st^ Progression | 1.124 | 0.314 | 1.933 | <0.001 |
| After 1^st^ Progression | 1.287 | 0.147 | 2.428 | 0.030 |
| Change Δ | Mean Change in Tumor Progression Rate (cm^2^/month) | Lower 95% CL | Lower 95% CL | P-value |
| Δ_(After – Prior)_ | 0.164 | -1.174 | 1.502 | 0.802 |

CL = confidence limit.

† P-value is for the testing of the null hypothesis that the mean tumor progression rate is equal to 0.
